# *Gnathopalystesaureolus* (He & Hu, 2000): new combination for *Pseudopodaaureola* (Araneae, Sparassidae), with the first description of the female from Hainan Island, China

**DOI:** 10.3897/zookeys.817.29868

**Published:** 2019-01-15

**Authors:** Wei Ding, Yang Zhong, Jie Liu

**Affiliations:** 1 The State Key Laboratory of Biocatalysis and Enzyme Engineering of China, Centre for Behavioural Ecology and Evolution, School of Life Sciences, Hubei University, Wuhan 430062, Hubei, China Hubei University Wuhan China; 2 School of Nuclear Technology and Chemistry & Biology, Hubei University of Science and Technology, Xianning 437100, Hubei, China Hubei University of Science and Technology Xianning China

**Keywords:** Biodiversity, Hainan, huntsman spiders, taxonomy

## Abstract

The taxonomic status of *Pseudopodaaureola* (He & Hu, 2000) is revised based on the re-examination of the type specimen and fresh material collected from the type locality. The cheliceral dentition, with a large denticle field between two anterior and three posterior teeth, the male palps with simple RTA arising distally, narrow, not filiform embolus and sheet-like membranous conductor, and the female epigyne with a visible median septum indicate that the species does not belong to *Pseudopoda* or to the originally assigned genus, *Heteropoda*. Based on these characters, the species is here transferred to *Gnathopalystes*. The male is redescribed and the female is described and illustrated for the first time.

## Introduction

*Heteropodaaureola* was first described in the genus *Heteropoda* Latreille, 1804, based on male specimens from Jianfengling Mountain, Hainan Province, China by He and Hu (2000). Jäger (2014) transferred it to *Pseudopoda* Jäger, 2000 based on the original description and illustrations, which showed an RTA with a bifid apex arising medially from the tibia. However, the author pointed out that a definite characterization would only be possible with the examination of type material or material from the type locality.

Recently, the authors examined material from Jianfengling Mountain (type locality of *P.aureola*) and found specimens which, when compared to the holotype, were confirmed as *P.aureola*. However, the presence of male palps with simple RTA, arising distally (medially or basally in *Pseudopoda*), narrow, not filiform embolus (broadened and flattened in *Pseudopoda*, filiform in *Herteropoda*), sheet-like membranous conductor (sheath-like in *Herteropoda*) and female epigyne with visible median septum (inconspicuous in *Pseudopoda*) indicated that this species did not belong to either *Heteropoda* or *Pseudopoda*. When comparing this material to that of other species recorded from China, the authors noted a strong similarity between *P.aureola* and *Gnathopalystestaiwanensis* Zhu & Tso, 2006 (described from Taiwan and recorded in China) which suggested that the species might actually belong to *Gnathopalystes*.

Rainbow (1899) established *Gnathopalystes* to include *G.ferox* Rainbow, 1899. Simon (1903) synonymized it with *Palystes* L. Koch, 1875, and this decision was generally accepted until Croeser (1996) revised *Palystes* and revalidated *Gnathopalystes* to accommodate the non-African species of the genus. Nevertheless, he diagnosed the genus using only habitus characters, such as eye arrangement and cheliceral dentition, because the type species, *G.ferox*, was known solely from an immature female. From that time on, most authors considered *Gnathopalystes* a valid genus and several new species were described (Jäger 1998; Zhu and Tso 2006; Saha and Raychaudhuri 2007; Jäger 2014). Currently, the genus includes nine species, most of which are distributed in Vanuatu, Solomon Is., Papua New Guinea, New Guinea, Taiwan, Indonesia, and Malaysia.

Although *Gnathopalystes* has not been revised, and most species are poorly described and illustrated, we decided to remove *P.aureola* from *Pseudopoda* and transfer it to *Gnathopalystes* based on characters provided by Croeser (1996) and Jäger (1998). *Gnathopalystesaureolus* comb. n. has a straight posterior eye row, wider than the recurved anterior eye row, with AME smaller than ALE, PLE equal or slightly larger than PME (PLE significantly larger than PME in *Heteropoda* and *Pseudopoda*). It also shows a large denticle field between the anterior and posterior teeth, which extends from the proximal teeth to the margin of the non-sclerotized arthrodial membrane (often close to the three anterior teeth in *Heteropoda* and *Pseudopoda*). In addition to the transfer, we provide a redescription of the male of *G.aureolus* comb. n. and describe the female for the first time.

## Materials and methods

Specimens were examined with an Olympus SZX16 stereomicroscope; details were further investigated with an Olympus BX51 compound microscope. All illustrations were made using an Olympus drawing tube. Epigynes were examined and illustrated after dissection from the spider bodies. Photos were made with a Canon G10 digital camera (14.7 megapixels) mounted on an Olympus SZX16 stereomicroscope. The digital images depicting the habitus and genital morphology are a composite of multiple images taken at different focal planes along the Z-axis and assembled using the software package Helicon Focus 3.10. Left palps are depicted unless otherwise stated. The illustration of schematic course of internal duct system follows Jäger (2000). Most hairs and macrosetae are usually not depicted in the palp and epigyne drawings. Positions of tegular appendages are given according to clock positions, based on the left male palp in ventral view. Measurements are given in millimeters.

Leg measurements are shown as: total length (femur, patella, tibia, metatarsus, tarsus). Number of spines is listed for each segment in the following order: prolateral, dorsal, retrolateral, ventral (in femora and patellae ventral spines are absent and fourth digit is omitted in the spination formula). Abbreviations follow Zhong et al. (2017, 2018):

**ALE** anterior lateral eyes;

**AME** anterior median eyes;

**AW** anterior width of prosoma;

**CH** clypeus height;

**FE** femur;

**Mt** metatarsus;

**OL** opisthosoma length;

**OW** opisthosoma width;

**Pa** patella;

**PH** prosoma height;

**PL** prosoma length;

**PLE** posterior lateral eyes;

**PME** posterior median eyes;

**Pp** palpus;

**PW** prosoma width;

**Ta** tarsus;

**Ti** tibia. I, II, III, IV–legs I to IV.

Abbreviations for the collection depositories:

**HBU**Hubei University, Wuhan, China;

**MTJ** Museum of Tianjing, Tianjing, China

## Taxonomy

### Family Sparassidae Bertkau, 1872

#### Genus *Gnathopalystes* Rainbow, 1899

##### Gnathopalystes aureolus

Taxon classificationAnimaliaAraneaeSparassidae

(He & Hu, 2000) comb. n.

**Figure 1.**
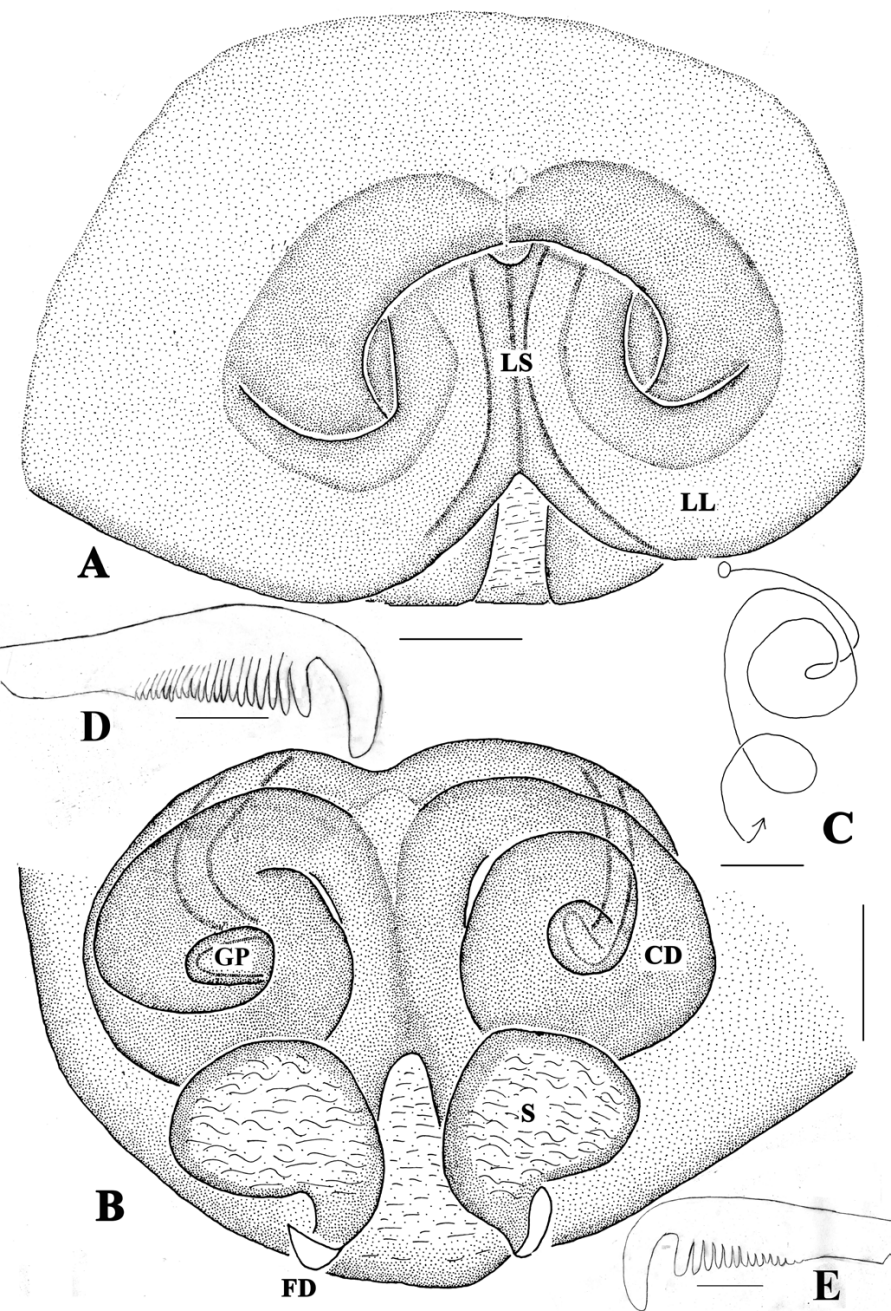
*Gnathopalystesaureolus* (He & Hu, 2000) comb. n. **A** Epigyne, ventral view **B** Vulva, dorsal view **C** Schematic course of internal duct system, dorsal view **D–E** Female tarsal claws of leg I (**D** prolateral **E** retrolateral). Abbreviations: C – conductor; CD – copulatory duct; FD – fertilization duct; GP – glandular projection; LL – lateral lobes; LS – lobal septum. Scale bars: 0.2 mm (**A–C**); 0.1mm (**D, E**).

**Figure 2.**
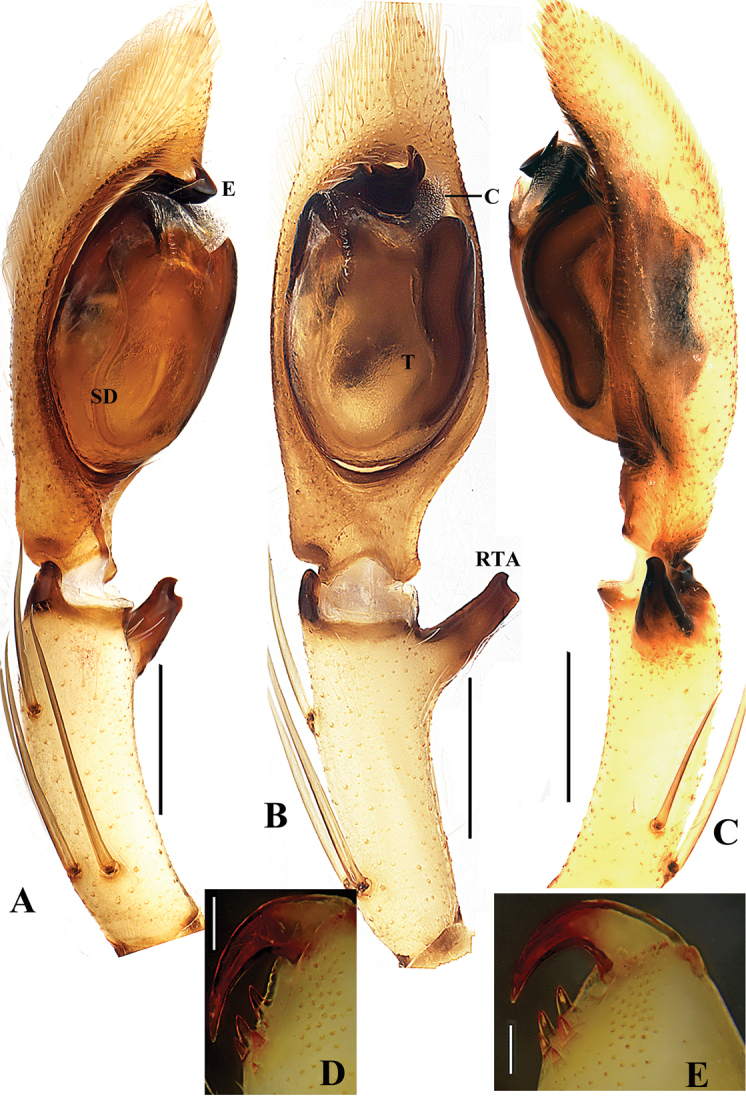
*Gnathopalystesaureolus* (He & Hu, 2000) comb. n. **A–C** Left male palp (**A** prolateral **B** ventral **C** retrolateral) **D** Male left cheliceral teeth, ventral view **E** Female left cheliceral teeth, ventral view. Abbreviations: C – conductor; E – embolus; RTA – retrolateral tibial apophysis; SD – sperm duct; T – tegulum. Scale bars: 0.2 mm.

**Figure 3.**
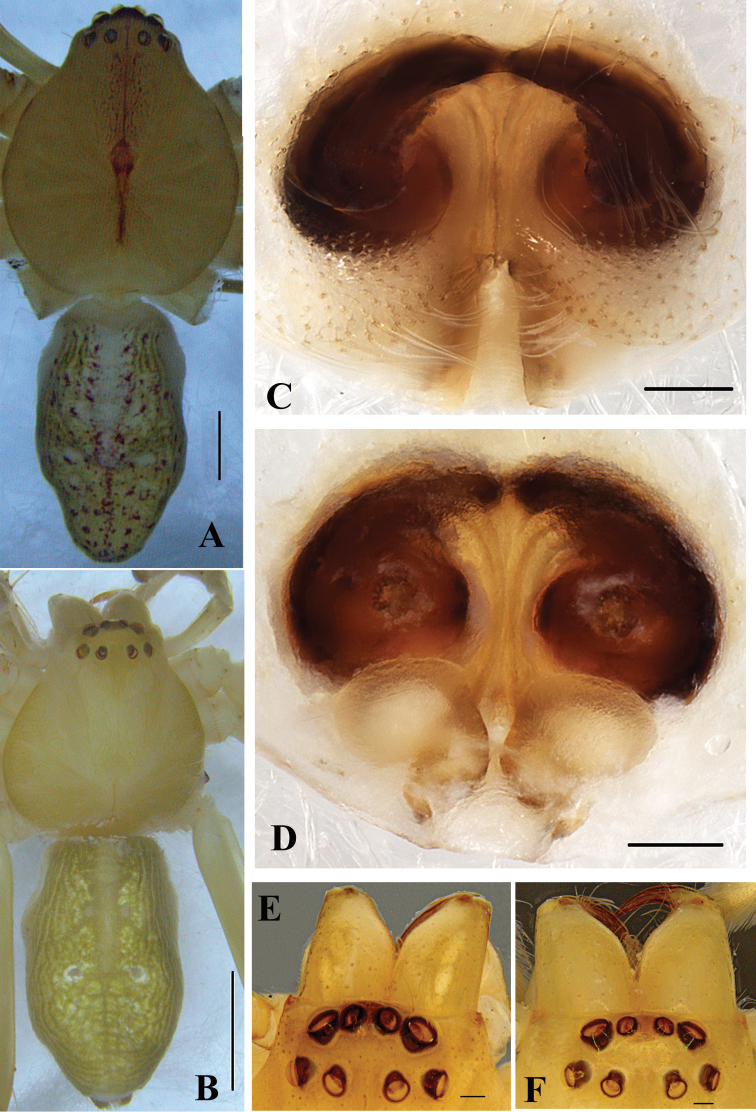
*Gnathopalystesaureolus* (He & Hu, 2000) comb. n. **A** Male habitus, dorsal view **B** Female habitus, dorsal view **C** Epigyne, ventral view **D** Vulva, dorsal view **E** Male eyes, dorsal view **F** Female eyes, dorsal view. Scale bars: 1mm (**A, B**); 0.2 mm (**C–F**).

**Figure 4.**
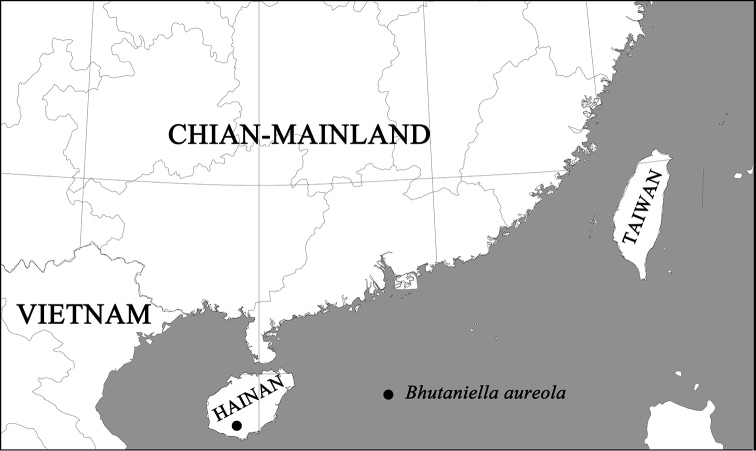
Collection locality of *Gnathopalystesaureolus* comb. n. in Hainan Island, China.

Figures 1 , 2 , 3 , 4

Heteropoda aureola He & Hu, 2000: 17, figs 1–2 (holotype male from Jianfengling Mountain, Hainan, China, deposited in MTJ, examined).
Pseudopoda aureola Jäger, 2014: 184 (transferred from Heteropoda).

###### Material examined.

1 male (holotype, MTJ), Jianfengling Mountain, Hainan Island, China, 8 April 1980, Shengli Liu leg.; 1 male and 1 female (HBU), Jianfengling Mountain [22°37.93'N, 120°63.29'E, 560 m], Hainan Island, China, 9 June 2013, Fengxiang Liu leg.

###### Diagnosis.

Females of *G.aureolus* comb. n. resemble those of *G.taiwanensis* in eye arrangement (straight posterior row and recurved anterior row, with posterior row wider than anterior, AME smaller than ALE), cheliceral dentition (large denticle field between two anterior and three posterior teeth), and epigyne (with coiled copulatory ducts). They differ by the epigyne with lateral lobes contiguous (separated in *G.taiwanensis*), anterior margin of lobal pockets distinct (absent in *G.taiwanensis*) and left part of vulva connected to the right part (separated in *G.taiwanensis*). The females of *G.taiwanensis* and *G.aureolus* comb. n. can be distinguished from other *Gnathopalystes* species by the three posterior teeth on the retromargin of cheliceral fang furrow (four to five posterior teeth in other *Gnathopalystes* species). Males can be distinguished by the palp with clavate and straight RTA (RTA broad at base, tapering to apex, and bent in *G.kochi* (Simon, 1880), the only other known male to date) (Figs 1, 2, 3C–F).

###### Description.

**Male**. Measurements: PL 4.26, PW 3.50, AW 1.37, PH 0.88, OL 4.28, OW 2.20. Eyes: AME 0.15, ALE 0.26, PME 0.18, PLE 0.21, AME–AME 0.20, AME–ALE 0.11, PME–PME 0.38, PME–PLE 0.25, AME–PME 0.45, ALE–PLE 0.30, CHAME 0.24, CHALE 0.12. Leg and palp measurements: Pp 5.15 (1.62, 0.79, 1.31, -, 1.43), I 22.77 (5.86, 1.11, 6.79, 7.08, 1.93), II 24.66 (7.00, 1.41, 6.86, 7.66, 1.73), III 17.49 (5.54, 1.20, 4.94, 4.45, 1.36), IV 21.35 (6.76, 1.22, 5.75, 6.04, 1.58). Leg formula: II-I-IV-III. Spination: Pp 120, 101, 3011; Fe I 223, II 323, III 222, IV 322; Pa I-IV 101; Ti I 2026, II 2025, III-IV 2024; Mt I-IV 2024. Cheliceral furrow with large denticle field extending from proximal teeth to the margin of the non-sclerotized arthrodial membrane, with approximately 18 denticles. Promargin of cheliceral furrow with two teeth, the distal one significantly larger than proximal one, retromargin with three teeth, the two distal ones similar sized, the proximal one significantly larger (Figure 2D). Dorsal shield of prosoma generally yellowish brown without distinct pattern, with a reddish longitudinal line medially, with a heavy reddish fovea and faint brown marks. Eye region slightly darker, eye borders dark. Sternum bright brown. Labium, gnathocoxae and chelicerae bright brown with orange margins. Legs and pedipalps bright brown. Opisthosoma yellowish-brown colored with dorsal pattern of scattered reddish marks (Figure 3A). Palp as in diagnosis. Tibia slightly shorter than cymbium with three prolateral, one dorsal, and one retrolateral spine. Cymbium with dense long hairs. Cymbial scopula distinct, elliptical and located on distal half of cymbium. Tegulum significantly large, almost occupying four-fifths of the alveolus. Subtegulum not visible in ventral view. Sperm duct U-shaped, tapering. Conductor irregular, with sharp tip in prolateral view, arising from tegulum at 11-o’clock-position. RTA simple, unbranched, arising distally from tibia (Figs 2A–C).

**Female**. Measurements: PL 3.95, PW 3.93, AW 1.90, PH 0.68, OL 5.24, OW 3.00. Eyes: AME 0.15, ALE 0.23, PME 0.17, PLE 0.2, AME–AME 0.25, AME–ALE 0.11, PME–PME 0.48, PME–PLE 0.27, AME–PME 0.49, ALE–PLE 0.36, CHAME 0.25, CHALE 0.10. Leg and palp measurements: Pp 3.93 (1.05, 0.77, 0.99, -, 1.12), I 20.28 (5.60, 1.72, 6.05, 5.51, 1.40), II 20.10 (5.95, 1.70, 5.81, 5.25, 1.39), III 13.28 (4.32, 0.97, 3.81, 3.17, 1.01), IV 16.37 (4.85, 1.03, 4.18, 5.03, 1.28). Leg formula: I-II-IV-III. Spination: Pp 202, 101, 2121, 2021; Fe I 023, II 010, III-IV 121; Pa I 000, II-IV 000; Ti I III- IV 2014, II 1013; Mt I-IV 2024. Cheliceral furrow as in male, with approximately 23 denticles (Figure 2E). Dorsal shield of prosoma generally pale brown without distinct pattern. Eye region slightly darker, eye borders dark. Sternum, labium, gnathocoxae and legs as in male. Opisthosoma yellowish-brown colored with two small white round marks medially and laterally (Figure 3B). Epigyne as in diagnosis. Anterior bands absent. Lateral lobes fused. Lobal septum wide, significantly short. Copulatory ducts coiled, with two small glandular projections, without integument. Fertilization ducts arising posterolaterally, widely separated (Figs 1, 3C–D).

###### Distribution.

China (Hainan) (Fig. 4).

## Supplementary Material

XML Treatment for Gnathopalystes aureolus
